# Transcriptome Sequencing and Comparative Analysis of Amphoteric ESCs and PGCs in Chicken (*Gallus gallus*)

**DOI:** 10.3390/ani10122228

**Published:** 2020-11-27

**Authors:** Kai Jin, Jing Zhou, Qisheng Zuo, Jiuzhou Song, Yani Zhang, Guobing Chang, Guohong Chen, Bichun Li

**Affiliations:** 1 Key Laboratory of Animal Breeding Reproduction and Molecular Design for Jiangsu Province, College of Animal Science and Technology, Yangzhou University, Yangzhou 225009, China; jinkai0621@163.com (K.J.); zhoujing980111@163.com (J.Z.); 006664@yzu.edu.cn (Q.Z.); ynzhang@yzu.edu.cn (Y.Z.); gbchang@yzu.edu.cn (G.C.); ghchen@yzu.edu.cn (G.C.); 2 Institutes of Agricultural Science and Technology Development, Yangzhou University, Yangzhou 225009, China; 3 Joint International Research Laboratory of Agriculture and Agri-Product Safety of Ministry of Education of China, Yangzhou University, Yangzhou 225009, China; 4 Animal & Avian Sciences, University of Maryland, College Park, MD 20741, USA; songj88@umd.edu

**Keywords:** differentially expressed genes, transcriptome analysis, amphoteric ESCs, amphoteric PGCs, chicken (*Gallus gallus*)

## Abstract

**Simple Summary:**

The study of chicken embryonic stem cells (ESCs) and primordial germ cells (PGCs) showed the potential application of developmental biology and translational medicine. However, the difference between amphoteric ESCs and PGCs is still elusive, limiting the accuracy of correlative research. In this paper, chicken amphoteric ESCs and PGCs were isolated, separated, and sequenced to explore their dynamic transcriptomes. Our results provide a knowledge base of transcriptomes in chicken amphoteric ESCs and PGCs, which will help other researchers interested in studying relative biological processes.

**Abstract:**

Chicken (*Gallus gallus*) pluripotent embryonic stem cells (ESCs) and primordial germ cells (PGCs) can be broadly applied in the research of developmental and embryonic biology, but the difference between amphoteric ESCs and PGCs is still elusive. This study determined the sex of collected samples by identifying specific sex markers via polymerase chain reaction (PCR) and fluorescence activated cell sorter (FACS). RNA-seq was utilized to investigate the transcriptomic profile of amphoteric ESCs and PGCs in chicken. The results showed no significant differentially expressed genes (DEGs) in amphoteric ESCs and 227 DEGs exhibited in amphoteric PGCs. Moreover, those 227 DEGs were mainly enriched in 17 gene ontology (GO) terms and 27 pathways according to Kyoto Encyclopedia of Genes and Genomes (KEGG) analysis. Furthermore, qRT-PCR was performed to verify RNA-seq results, and the results demonstrated that Notch1 was highly expressed in male PGCs. In summary, our results provided a knowledge base of chicken amphoteric ESCs and PGCs, which is helpful for future research in relevant biological processes.

## 1. Introduction

Chicken (*Gallus gallus*) is a classical model in developmental and embryonic biology [1]. For decades, chickens have played a vital role in animal research as alternatives and outbred experimental species to humans to compensate for ethical constraints [2,3,4]. It works especially well when isolating and gaining a considerable number of pluripotent embryonic stem cells (ESCs) and primordial germ cells (PGCs) is much easier and convenient in chickens than other species due to the in ovo embryonic development [5,6]. This unique advantage accelerates the application of chicken ESCs and PGCs in studying the mechanism of human germ cell development and differentiation [7,8]. Many groups have recently focused on the practical application of chicken ESCs and PGCs in human medicine-related research, including germ cell tumor and drug target screening [9,10].

In chickens, ESCs have been derived from cultures of chicken blastoderms taken from stage X-XII and PGCs can be obtained from the genital ridge (the precursor of gonad) at stage 28–30 embryos [6,11]. ESCs (blastoderms) represent the earliest accessible post-laying developmental stage prior to primitive streak formation and gastrulation; PGCs (genital ridge) represent the time when gonads are still morphologically identical in each sex (“bipotential”) [12,13]. These ESCs are positive for telomerase activity, alkaline phosphatase, and the antigen SSEA-1 or pluripotent genes (SOX2, OCT4 and NANOG), while PGCs exhibit the ability of migration and positiveness for the antigen SSEA-1 or germ cell gene (CKIT and CVH) [14,15]. Moreover, PGCs are the precursor of germline cells that differentiate into male or female gametes that give rise to progeny [6,16]. Although many studies focus on chicken ESCs and PGCs and the collection method has been established, no research has been reported to reveal the difference between amphoteric ESCs and PGCs.

As a powerful way to describe dynamic changes in gene expression, transcriptome analysis provides crucial clues to help understand the process of embryogenesis and embryonic development, and it has been used for studying chicken germ cells and embryogenesis [17]. Studies on male and female differences in chickens have traditionally focused on tissue levels (blastoderm or gonad) instead of comprehensive transcriptome dynamics in amphoteric ESCs and PGCs [18,19,20].

Therefore, in this study, RNA-seq was performed to investigate the profile of chicken amphoteric ESCs and PGCs through characterizing differentially expressed genes (DEGs), significant gene ontology (GO) terms, and Kyoto Encyclopedia of Genes and Genomes (KEGG) pathways enriched in amphoteric ESCs and PGCs. This work will provide a better understanding of the expression pattern of chicken amphoteric ESCs and PGCs, which will aid in further studies of relative biological processes.

## 2. Materials and Methods

### 2.1. Materials and Ethics

The chicken eggs were collected from the Rugao Yellow Chicken (Poultry Institute, Chinese Academy of Agricultural Sciences, Yangzhou, China). All eggs were incubated under the environment of 37 °C and 75% relative humidity for 4.5 days. The animal experiments were approved by the Institutional Animal Care and Use Committee of the Yangzhou University Animal Experiments Ethics Committee (Permit Number: SYXK [Su] IACUC 2012-0029).

All experimental procedures were performed in accordance with the Regulations for the Administration of Affairs Concerning Experimental Animals approved by the State Council of the People’s Republic of China.

### 2.2. Determination of Sex of Chicken Embryos by PCR

Cell or tissue samples were collected and used as PCR templates. CHD1 gene on the sex chromosome (Z/W) was amplified by PCR using Mighty Amp DNA Polymerase. The gel electrophoresis was performed to validate the PCR amplified products. The band of PCR products amplified was at 580 bp, and the bands in females (ZW) were at 580 and 423 bp.

The primer sequences were as follows:

CHD-Forward: CTGCGAGAACGTGGCAACAGAGT;
CHD-Reward: ATTGAAATGATCCAGTGCTTG.

### 2.3. Cells Isolation and Culture

Isolation and culturing of amphoteric ESCs and PGCs were described in a previous report [21]. The medium contained 43.5 mL Knockout-DMEM (Gibco, New York, NY, USA, 10829018), 100 μL gentamicin (Solarbio, Beijing, China, G8170), 0.2 μL β-mercaptoethanol (Sigma, MI, USA, M3148), 200 μL non-essential amino acids (Sigma, MI, USA, M7145), 1 mL chicken serum (Gibco, New York, NY, USA, 16110-082), 100 μL SCF (Sigma, MI, USA, 300-07-10), 100 μL bFGF (Sigma, MI, USA, F0291), 50 μL LIF (Millipore, MA, USA, ESG1106), and 500 μL penicillin (Solarbio, Beijing, China, P1400-100). The induction medium was Dulbecco’s Modified Eagle Medium (DMEM) supplemented with10 mM RA (Solarbio, Beijing, China, IR0060) and 15% Fetal Bovine Serum (FBS) (Gibco, New York, NY, USA, 26140).

### 2.4. Fluorescence Activated Cell Sorter (FACS)

Cells harvested from amphoteric ESCs and PGCs were blocked with blocking buffer (Phosphate Buffered Saline containing 10% fetal bovine serum) (Gibco, New York, NY, USA, 10270-106) for 2 h at 37 °C. Samples were incubated with antibodies against cell surface epitopes (SSEA-1, abcam, Cambridge, UK, ab16285,1:100; SOX2, abcam, Cambridge, UK, ab93689,1:100; CKIT, Thermo Fisher Scientific, Shanghai, China, 14-1172-81,1:100) at 4 °C overnight and washed with PBS containing 0.1% Tween-20 (Solarbio, Beijing, China, T8220) three times, followed by fluorescence coupled secondary antibody (Goat Anti-Rabbit IgG FITC Conjugated, CWBIO, Shanghai, China, CW0114S, 1:100; Goat Anti-Mouse IgG H & L (TRICT), abcam, Cambridge, UK, ab6786, 1:100) incubation at 37 °C for 2 h. Then, the cells were washed with PBS containing 0.1% Tween-20 three times. The staining signal was analyzed by fluorescence activated cell sorter (FACS) LSRFortessa (BD Biosciences, San Jose, CA, USA) with a minimum of 10,000 events in each experiment, and sorted cells were collected for further experiments.

### 2.5. RNA Extraction and Sequencing

RNA was extracted with TRIzol reagent (Invitrogen, Carlsbad, CA, USA) according to the manufacturer’s instructions. To effectively remove the genomic DNA, we added RNase-free DNase I (Takara, Dalian, China) to the reaction mixture for at least 10 min. The extracted RNA was quantified with the Nanodrop system (Thermo, Wilmington, DE, USA), and the fragment size distribution was checked by 1.5% agarose gel electrophoresis. RNA concentration was assessed using a Thermo NanoDrop2000TM spectrophotometer (Thermo Fisher Scientific, Wilmington, DE, USA), and the RNA integrity number (RIN) was determined using an Agilent 2100 Bioanalyzer (Agilent Technologies, Santa Clara, CA, USA) (Table 1). The extracted RNA was stored for reverse-transcription and sequencing. The RNA Libraries pools of four kinds of cells were established following the protocol of Illumina mRNA-seq with 50 ng RNA, and the experiments were performed by the Shanghai OE Biotech Co., Ltd. (Shanghai, China).

### 2.6. Differential Expression Analysis and Functional Enrichment

Filtering and quality control checks of raw reads from RNA-seq were done by FastQC [22]. The clean reads were mapped to reference sequences using SOAP2 [23]. The gene expression levels were quantified by calculating the RPKM (Reads Per kb transcriptome per Million reads) values. The gene expression pattern and PCA analyses were performed on R [24]. The expression of genes with a fold change > 2 and FDR < 0.001 were filtered as differentially expressed genes (DEGs). The functional analysis of DEGs was carried out by GO analysis and KEGG pathway enrichment analysis using DAVID [25].

### 2.7. Quantitative Real-Time Polymerase Chain Reaction (qRT-PCR)

Tissues and cells were homogenized in TRIzol Reagent, and total RNA was isolated according to the manufacturer’s instruction (QIAGEN, Beijing, China, DP424). qRT-PCR was performed using the FastKing One-Step RT-PCR Kit with SYBR green (QIAGEN, Beijing, China, KR123). mRNA levels of related genes were determined by CFX-Connect Real-Time PCR detection system (BIO-RAD, California, USA, 7500fast). Results were represented as quantification normalized to relative housekeeping genes (*β-Actin*) using the 2-ΔΔCt method. The sequences of qRT-PCR primers are listed in Table 2.

## 3. Results

### 3.1. Amphoteric ESCs and PGCs Confirmation

**Figure 1:**
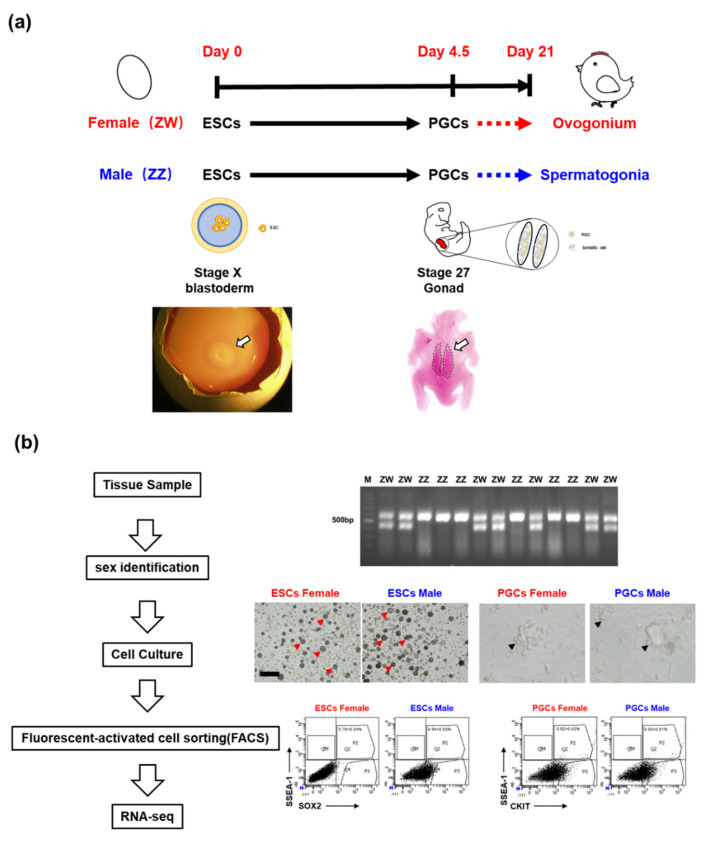
Experimental design and confirmation of amphoteric ESCs and PGCs in chicken. (**a**) Tissue collection of the amphoteric blastoderm (for ESCs) and gonads (for PGCs); (**b**) experimental design of RNA-seq analysis (**left**) and amphoteric ESCs and PGCs confirmation by PCR (**right-top**), morphology (**right medium**, the red arrow points to typical ESCs clones, and the black arrow points to emblematic PGCs mass), and fluorescence activated cell sorter (FACS) (**right bottom**).

For sample preparation for transcriptome analyses, the blastoderms and gonads were collected from incubated chicken eggs at day 0 and day 4.5 (Figure 1a). The sex of collected tissues was identified by PCR as described previously. PCR products with two bands (600 and 450 bp) were observed in tissues collected from females (ZW) and only one band (600 bp) in males (ZZ). In total, ESCs were isolated from 9699 eggs, including 4845 females and 4854 males; PGCs were obtained from 3150 eggs, including 1594 males and 1556 females. Samples of same-sex eggs from each stage were collected for isolation and culturing. The result showed that no morphological differences between the cultured amphoteric ESCs and PGCs. To further confirm the cell type, different cell surface markers (SSEA-1& SOX2 for ESCs; SSEA-1 &CKIT for PGCs) were used to isolate different types of cells by the FACS. FACS results showed the efficiency is 0.78+/−0.04% (ESCs female), 0.94+/−0.03% (ESCs male), 0.92+/−0.02% (PGCs female), and 0.93 + 0.01% (PGCs male), respectively (Figure 1b).

### 3.2. Transcriptome Characterization of Amphoteric ESCs and PGCs in Chickens

**Figure 2:**
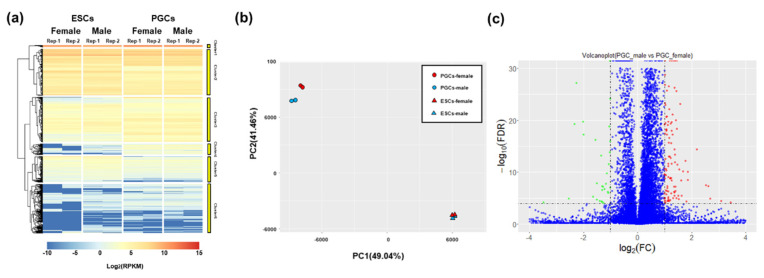
Transcriptome characterization of amphoteric ESCs and PGCs in chicken. (**a**) Heatmap showing differentially expressed genes (DEGs) in amphoteric ESCs and PGCs. Blue represents weakly expressed genes, and red represents highly expressed genes; (**b**) principal component analysis for eight samples. The first two principal components are displayed on the graph; (**c**) volcano plot where the *x*-axis represents the level of differential expression and the *y*-axis shows significant differences in expression as negative log values. The horizontal line is the threshold of corrected FDR < 0.001(−log10(FDR) > 3). In ovaries, downregulated genes are indicated by green dots, upregulated genes in ovaries are indicated by red dots, and other genes are indicated by blue dots.

Eight sequencing libraries were constructed for the Illumina platform with two biological repeats to obtain the genome-wide gene expression profile of amphoteric ESCs and PGCs. In total, 36,221 transcripts could be detected in combined transcriptomes, and gene expression was quantified by calculating RPKM values. A heatmap illustrating the hierarchical clustering of RPKM values was generated to visualize the overall gene expression pattern (Figure 2a). The PCA analysis was utilized to explore gene expression patterns among amphoteric ESCs and PGCs, revealing different patterns between ESCs and PGCs. Moreover, it showed that amphoteric ESCs clustered together closely, while amphoteric PGCs were relatively dispersed, indicating amphoteric ESCs exhibit similar patterns, but PGCs exhibit different patterns (Figure 2b). Differential analysis of transcriptome data demonstrated no DEGs in amphoteric ESCs, while 227 DEGs were found in amphoteric PGCs (Figure 2c and Figure S1). Among 227 DEGs, 52 DEGs were highly expressed in female PGCs and 172 in male PGCs (Table S1). Furthermore, no known or putative regulator of gonadal sexual determination and differentiation was identified among 227 DEGs, which implied that major components of sex determination and differentiation pathways are activated after this stage, but the amphoteric PGCs initiated the differentiated expression.

### 3.3. Enrichment Analysis of DEGs in Amphoteric PGCs in Chickens

**Figure 3:**
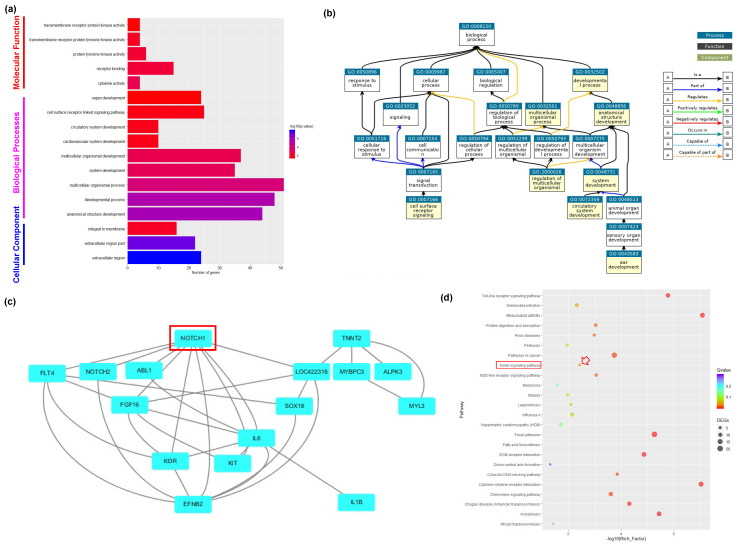
Enrichment analysis of DEGs in amphoteric PGCs in chicken. (**a**) Significant GO terms in amphoteric PGCs; (**b**) the directed acyclic graph (DAG) of biological processes (BP) GO term in amphoteric PGCs; (**c**) prediction of protein–protein interaction (PPI) relationship of DEGs in amphoteric PGCs; (**d**) the KEGG enrichment analysis of the DEGs in amphoteric PGCs. The red arrow points to the Notch signal.

The gene ontology database [26] was used to perform functional annotation on DEGs transcripts’ three components, including biological processes (BP), molecular function (MF), and cellular component (CC). The clusterProfiler R package was used to analyze GO enrichment of DEGs transcripts. GO terms (corrected *p*-value < 0.05) were considered significantly enriched. The 17 significantly enriched GO terms were annotated as biological processes (9; 52.9%), cellular component (3; 17.64%), and molecular function (5; 29.41%), as shown in Figure 3a and Table S2. Interestingly, almost all GO terms in biological processes (8/9) were sub-terms of the developmental process, which indicated the developmental-difference process is active in amphoteric PGCs (Figure 3b). Moreover, we concluded that Notch1 is a key gene in developmental process terms according to all genes’ interaction prediction analysis (Figure 3c). Pathway significant enrichment analysis was performed to identify host genes involved in major biochemical metabolic pathways and signal transduction pathways in KEGG database [27]. The clusterProfiler R package was used to analyze the statistical enrichment of genes differentially expressed in KEGG pathways. Specifically, 24 KEGG terms were significantly enriched in chicken amphoteric PGCs (Figure 3d and Table S3). In addition, the Notch pathway was also significantly enriched in amphoteric PGCs, which is consistent with the interaction prediction analysis results.

### 3.4. Validation of Transcriptome Data by Real-Time qRT-PCR and Notch1 Specific Expression in Male PGCs

**Figure 4:**
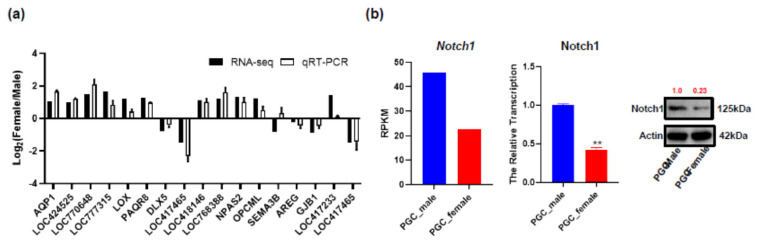
Validation of transcriptome data by real-time qRT-PCR and Notch1 specific expression in male PGCs. (**a**) Expression comparisons of selected genes detected by RNA-Seq and qRT-PCR between amphoteric ESCs and PGCs. The *y*-axis shows log2 (fold differences) determined by RNA-Seq and qRT-PCR. The experiments were repeated three times and provided consistent results. The mean values and error bars were obtained from three biological and three technical replicates; (**b**) the expression of Notch1 in amphoteric PGCs: the RPKM values of RNA-seq result (left), the expression level of RNA (RT-qPCR, medium) and protein (Western blot, right) results of amphoteric PGCs. All experiments were repeated three times and provided consistent results. The mean values and error bars were obtained from three biological and three technical replicates. The red number in Western Blot presents normalized intensity. **: means differ significantly (*p* ≤ 0.05)

A total of 17 genes were selected to validate the result obtained from RNA-Seq data (Figure 4a). Consistent qPCR results indicated the reliability of RNA-Seq data and quantified gene expression accuracy in amphoteric PGCs.

High expression of Notch1 was found in male PGCs at both mRNA and protein levels, which demonstrated the Notch1 is a differential expression factor in amphoteric PGCs (Figure 4b).

## 4. Discussion

In this study, we conducted the transcriptomic gene expression analysis of amphoteric ESCs and PGCs in chickens. No genes were differentially expressed in amphoteric ESCs; however, differentially expressed genes were identified in amphoteric PGCs and also found to be highly enriched in significant GO terms and KEGG pathways. Moreover, we confirmed that Notch1 is highly expressed in male PGCs.

Genetic sex determination in mammals and birds occurs at fertilization with the differential inheritance of sex chromosomes. However, sex determination and differentiation are complicated processes that occur during embryonic development [28,29]. As omnipotent or pluripotent cells, ESCs, especially chicken ESCs, have been reported to resemble mice ESCs, which retain the stage of naive and primed [30,31,32]. Regarding mice, expression patterns in males and females vary in germ cells, which indicated that sexual dimorphisms occur in mouse ESCs [33]. However, our finding showed that chicken amphoteric ESCs share a great number of genes and have similar expression patterns, which indicates the homogeneity and highly undifferentiated stages of chicken amphoteric ESCs. Although the sex of both chickens and mice is controlled by sex chromosomes (W/Z or X/Y system), the sex determination in chickens is still elusive because of the absence of X chromosome inactivation [34,35,36]. We propose that might exhibit a potential specific mechanism cause the different expression patterns in amphoteric ESCs between chickens and mice.

Genital ridges are precursor organs of gonads (ovary and testis) containing PGCs and somatic cells [37,38,39]. In gonads, the transition of germ cells from PGCs to sperm cells or eggs is not determined by the genotype but by the somatic cell type of gonads [40,41]. The RNA-seq data revealed sexually dimorphic gene expression in gonad (genital ridge) tissues (day 4.5) before gonadal differentiation (day 6), including some sexual differentiation genes such as FOXL2 [19]. In this paper, the amphoteric PGCs initiate dimorphic gene expression, but these DEGs not including known or putative sex genes. Compared with gonad tissues at day 4.5, amphoteric PGCs represent lower heterogeneity, suggesting the uninitiated germ cell sex determination. A recent study has demonstrated that the amphoteric chicken PGCs could be induced by spermatogonial stem cells (SSCs) or oocytes via co-culture with sertoli cells or granulosa [42,43,44], which showed that PGC sex differentiation could be activated by somatic cells; this may explain different expression patterns of amphoteric gonad tissues with PGCs.

Furthermore, according to our gene ontology analysis, many DEGs are involved in the developmental process and KEGG pathways showed much cell-to-cell interaction and restructuring of the gonad into the morphologically distinct cytokine–cytokine receptor interaction, Toll-like receptor signaling pathway, focal adhesion, and ECM-receptor interaction. Chicken gonadal sexually dimorphic gene expression has been reported to be involved in ECM and cytoskeleton formation and regulation to facilitate testis or ovary formation [20]. Therefore, we conclude that amphoteric PGCs could interact with gonad somatic cells to drive the amphoteric morphological change in E4.5. Moreover, we identified the notch signal key factor, Notch1, is highly expressed in male PGCs and plays a critical role in the interaction network of DGEs. It is well known that Notch1 and notch signal are essential in cell–cell communication and further regulate embryonic development. In mice, Notch1 was reported to induce Sox9 expression during the early stages of embryo differentiation, and Sox9 is a crucial regulator through the process of masculinity [45]. Similarly, constitutive activation of Notch1 signaling in sertoli cells causes the exit of gonocytes from quiescence, indicating Notch1 promotes masculinity during sex differentiation [46]. In chickens, our previous study showed that the Notch1 and notch signal plays a crucial role in chicken PGC formation and production of spermatogonium [47]. Thus, our future studies will be focusing on investigating the effect of amphoteric PGCs and gonad somatic cells during sex differentiation and the functional role of Notch1 in amphoteric PGCs.

## 5. Conclusions

Chicken amphoteric ESCs and PGCs were isolated, separated, and sequenced to explore their dynamic transcriptomes. According to our results, amphoteric ESCs share many genes and exhibit similar expression patterns, implying that chicken amphoteric ESCs are homogenous and still in highly undifferentiated stages. In contrast, chicken amphoteric PGCs are heterogeneous and exhibit differentially expressed genes enriched in GO terms and the KEGG pathway involved in developmental processes. High expression of Notch1 in male PGCs might play a key role in amphoteric PGC differentiation. Overall, our results provide a knowledge base of transcriptomes in chicken amphoteric ESCs and PGCs, which would help other researchers interested in relevant biological processes.

## Figures and Tables

**Table 1:** The purity and competencies of RNA.

Cell Type	Gender	Sample Number	Concentration (μg/μL)	Purity		Volume (μL)	Total (μg)	28S/18S ^5^	Completeness (RIN ^6^)
				A260/280 ^3^	A260/230 ^4^				
ESCs **^1^**	Male	rep1	0.7313	1.85	1.43	25	18.28	2.2	9.3
		rep2	0.8399	1.92	1.47	35	29.40	1.5	9
	Female	rep1	0.9090	1.99	1.59	25	22.73	1.7	9.8
		rep2	0.6299	1.91	1.55	35	22.05	1.7	8.8
PGCs **^2^**	Male	rep1	1.1936	1.90	1.72	25	29.84	1.62	10
		rep2	0.8278	1.90	1.7	35	28.97	1.5	10
	Female	rep1	1.2127	1.91	1.78	25	30.32	1.8	10
		rep2	0.9808	1.99	1.93	35	34.33	1.9	10

^1^ embryonic stem cells; ^2^ primordial germ cells; ^3^ the ratio of absorbance at 260 and 280 nm; ^4^ the ratio of absorbance at 260 and 230 nm; ^5^ the ratio of 28S/18S ribosomal RNA; ^6^ RNA integrity number.

**Table 2:** The primers for qRT-PCR.

Gene Name	Primer Sequence (5′-3′)	
*AQP1*	F	AAATGTTCTGGAGGGCGGTG
	R	AAGCCAGCGAAACCTTCACG
*LOX*	F	ACTTGTTAGACGCCAGCTCG
	R	CGCGTATCGTCTGTAATACCCG
*NPAS2*	F	AGGGGAAGTCGTGCTGCTAT
	R	TGCACACGATGAACTCTGGC
*OPCML*	F	CCCTCATGTGCTTGGCCTTT
	R	TGTGATGCCCGTGATCTCCA
*PAQR8*	F	CTTCCAGAAGCACAACGAGGT
	R	CAAAGGCAGAGACCACGCAT
*LOC768388*	F	TAAAGGCGTGGCTACTGGGA
	R	TCCGTGTCCTGGCTTGTCTT
*LOC417233*	F	TATCCCGGCATCACAGGTGT
	R	GCAATACACACAGGCTGCCA
*LOC417465*	F	AACCGTGTGCTGCTTCAACT
	R	TGAACACAACTGCTGCCTGT
*LOC424525*	F	TGGGAGGTGACAGAGGCAAA
	R	CAACACCCGCAGCTGTACAT
*LOC418146*	F	AACAACGAGACGGGGCAAAG
	R	GGATCATGGCTTGCAGTGCT
*LOC770648*	F	TCTGAAAACTCCAGCACGCC
	R	GCAACCGCCTCCACAACTAT
*LOC777315*	F	CGCTGTTGCTGCGTGCCA
	R	CGCTTTCTGCGGGGACAGA
*LOC418840*	F	AGTTCGACTCTGCAGCTCCA
	R	AGGGTGGCATGCAGTACAGA
*DLX5*	F	GTGAGGATGGTGAACGGCAA
	R	AGGGCGAGGTATTGGGTCTT
*AREG*	F	TGGTGAACGCTGTGGTGAAC
	R	CCTGACCTGCACTATCACAATGA
*SEMA3B*	F	CGCACTTTGATGAGCTCCGT
	R	GGCCATGGTGTAAACGCAGA
*GJB1*	F	GTGCTCAACATGGCCGAGTT
	R	CAGCAGCTGGTTGATCTCGT
*β-actin*	F	CAGCCATCTTTCTTGGGTAT
	R	CTGTGATCTCCTTCTGCATCC

## Data Availability

The Illumina reads are available in the Sequence Read Archive (SRA) database at National Center for Biotechnology Information (NCBI) under study accession number PRJNA608148.

## Supplementary Materials

The following are available online at https://www.mdpi.com/2076-2615/10/12/2228/s1, Figure S1: The DEGs of amphoteric ESCs and PGCs in chicken, Table S1: The DEGs in amphoteric PGCs, Table S2: The significant GO terms in amphoteric PGCs, Table S3: The significant KEGG terms in amphoteric PGCs.
