# Supplementary figures and images for: Transcriptome Sequencing and Comparative Analysis of Amphoteric ESCs and PGCs in Chicken (Gallus gallus)
Source: Animals (Basel). 2020 Nov 27;10(12):2228. doi: 10.3390/ani10122228 (PMC7760303; doi:10.3390/ani10122228)

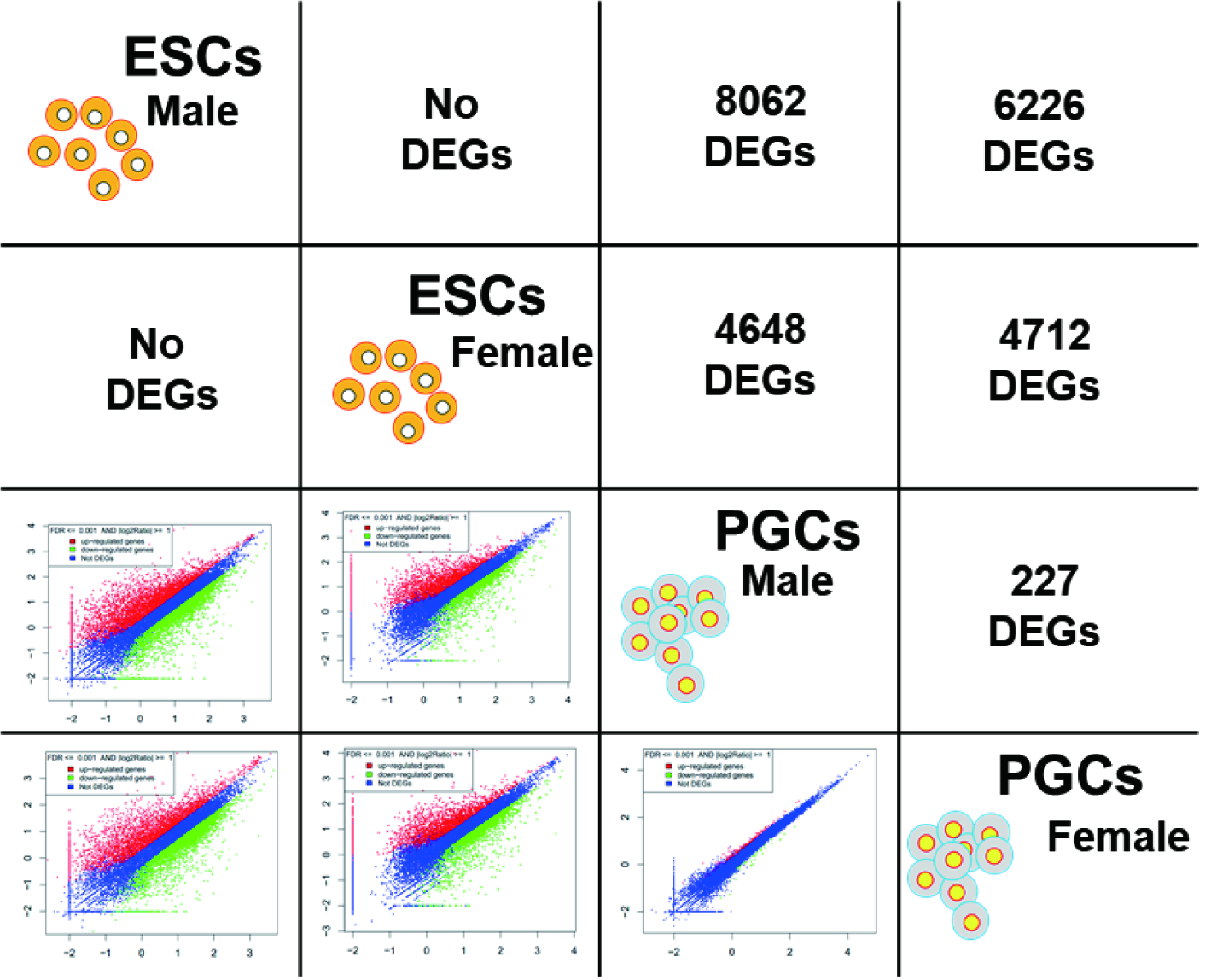

Supplement: Supplementary file 1 [file animals-10-02228-s001.zip › Fig S1.tif]
